# Optimal strategy of sEMG feature and measurement position for grasp force estimation

**DOI:** 10.1371/journal.pone.0247883

**Published:** 2021-03-30

**Authors:** Changcheng Wu, Qingqing Cao, Fei Fei, Dehua Yang, Baoguo Xu, Guanglie Zhang, Hong Zeng, Aiguo Song

**Affiliations:** 1 College of Automation Engineering, Nanjing University of Aeronautics and Astronautics, Nanjing, Jiangsu, China; 2 School of Aviation Engineering, Nanjing Vocational University of Industry Technology, Nanjing, Jiangsu, China; 3 School of Instrument Science and Engineering, Southeast University, Nanjing, Jiangsu, China; 4 Department of Mechanical Engineering, City University of Hong Kong, Hong Kong, China; Beihang University, CHINA

## Abstract

Grasp force estimation based on surface electromyography (sEMG) is essential for the dexterous control of a prosthetic hand. Nowadays, although increasing the number of sEMG measurement positions and extracting more features are common methods to increase the accuracy of grasp force estimation, it will increase the computational burden. In this paper, an approach based on analysis of variance (ANOVA) and generalized regression neural network (GRNN) for optimal measurement positions and features is proposed, with the purpose of using fewer measurement positions or features to achieve higher estimation accuracy. Firstly, we captured six channels of sEMG from subjects’ forearm and grasp force synchronously. Then, four kinds of features in time domain are extracted from each channel of sEMG. By combining different measurement position sets (MPSs) and feature set (FSs), we construct 945 data sets. These data sets are fed to GRNN to realize grasp force estimation. Normalized root mean square error (NRMS), normalized mean of absolute error (NMAE), and correlation coefficient (CC) between estimated grasp force and actual force are introduced to evaluate the performance of grasp force estimation. Finally, ANOVA and Tukey HSD testing are introduced to analyze grasp force estimation results so as to obtain the optimal measurement positions and features. We obtain the optimal MPSs for grasp force estimation when different FSs are employed, and the optimal FSs when different MPSs are utilized.

## Introduction

Prosthetic hands have irreplaceable significance for upper limb amputees. They can not only decorate missing limbs in appearance, but also restore body functions to a certain extent. Currently, several sEMG-based pattern recognition systems have been investigated for the control of prosthetic hands [1–4]. These systems capture sEMG from amputees’ residual arm, recognize the movement patterns, and control the movements towards the prosthetic hands. However, in the dexterous control of a prosthetic hand, both gestures and grasp force are critical.

In numerous studies, the process of hand grasp force estimation based on sEMG mainly involves signal collection (including sEMG and hand grasp force), signal pre-processing (including signal filtering, feature extraction, and so forth), and regression algorithm.

In terms of signal collection, the number of channels used varies from one to nearly 200. Wu et al. used two channels of sEMG to achieve grasp force estimation, and controlled a prosthetic hand with the estimation results [5]. Zhang et al. investigated pattern recognition and force estimation using four channels of sEMG from subject’s forearm through linear discriminant analysis and artificial neural network (ANN). They obtained the average error of force estimation, i.e., 10% [6]. Kim proposed a tensor algebra-based algorithm to predict the grasp force with respect to six channels of sEMG and the grasp posture [7]. Ban proposed a solution for estimating the direction and magnitude of the force applied on the object to be grasped using eight channels of sEMG of the forearm with convolutional neural network (CNN). His study achieved an accuracy of above 95% for directional estimation, and an NRMS of 7% for force magnitude estimation [8]. Ma adopted BPNN and gene expression programming (GEP) algorithm to realize grasp force prediction from 16 channels of sEMG [9]. Huang [10], Hu [11], and Zhang [12] employed a high-density array with 128 channels of sEMG electrodes to predict the muscle force, elbow-flexion force, and joint force, respectively. Chen et al. performed a cross-comparison of sEMG to a force method based on CNN and recurrent neural network (RNN) for multi degrees of freedom finger force prediction with high-density sEMG signals (160 channels) [13].

In terms of feature extraction, common features include time-domain, frequency-domain, and time-frequency-domain. The number of extracted features varies from one to dozens. Yokoyama et al. extracted root mean square from four channels of sEMG, and used neural networks with different numbers of hidden layers to estimate the hand grasp force. The correlations between predicted data and observed data were 0.840, 0.770, and 0.789 in the intrasession, intrasubject, and intersubject evaluations, respectively [14]. Yamanoi et al. extracted two features of mean absolute value and power spectrum from five channels of sEMG and performed simultaneous grip force estimation [15]. Baldacchino et al. extracted four features from EMG signals in NinaPro database, and applied them to a unified Bayesian framework to realize simultaneous force regression [16]. Kamavuako et al. investigated simultaneous and proportional estimation of force in two degrees of freedoms from intramuscular electromyography. In their work, five EMG features were extracted to feed ANN. Their results showed that the correlation coefficient between the estimated force and the actual force was 0.88 ± 0.05 with post processing [17]. In Yang’s work, ten features of the sEMG time domain were extracted and used as the input of a GEP-based model to estimate the grasp force [18]. Martinez et al. used linear regressors and the 10 transient features of the 16 channels of sEMG from subject’s forearm to estimate the grasp force. Their study reported an absolute error of 2.52% for the maximum voluntary force [19].

In terms of regression algorithm, neural network-based method is one of the most common methods. Yang et al. used three methods, local weighted projection regression, ANN, and support vector machine (SVM), to find the optimal regression relationship between sEMG and grasp force. Their experiment results indicated that SVM performed best among them [20, 21]. Xu et al. adopted CNN, long short-term memory, and their combination with 128 channels of sEMG to predict the muscle force generated in static isometric elbow flexion under three different circumstances. They obtained mean percentage root mean square errors of 9.07±1.29 and 8.67±1.14 in a subject-independent situation [22]. Cao et al. investigated hand grasp force prediction based on sEMG of forearm muscles and extreme learning machine (ELM), and compared the results of ELM with those of SVM and multiple nonlinear regression. They found that ELM possessed a relatively good accuracy and took less time, although SVM was effective for grasp force estimation in terms of accuracy [23].

The studies mentioned above used different channels of sEMG, sEMG features, and regression methods to realize hand force estimation. In most studies, accuracy is improved through using more channels of sEMG and extracting more features. However, more measurement positions and features mean a greater computational burden of the regression algorithm, which is unfavorable for the systems with limited processing capacity, such as some embedded real-time systems.

In this paper, to acquire high-accuracy estimation of the grasp force with less computation, we propose an optimal strategy of sEMG measurement position and feature for grasp force estimation based on ANOVA and GRNN.

## Materials and methods

**Fig 1:**
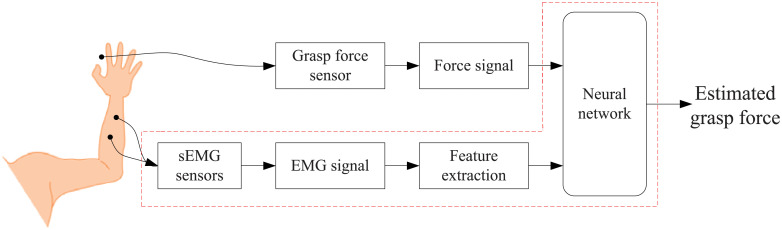
The structure of grasp force estimation.

Fig 1 shows the structure of grasp force estimation based on sEMG and neural network. In the procedure of model training, sEMG and grasp force are captured simultaneously. Then, several features are extracted from sEMG. Finally, the captured grasp force and sEMG features are regarded as the samples for network training. In grasp force estimation, only extracted sEMG features are imported to the trained neural network. The neural network will output the estimated grasp force.

### Experiment platfom

The experiment platform set up in this paper consists of an sEMG Armband, a grasp force sensor, a data collector, and a Microsoft foundation classes (MFC) based computer software.

#### sEMG armband

**Fig 2:**
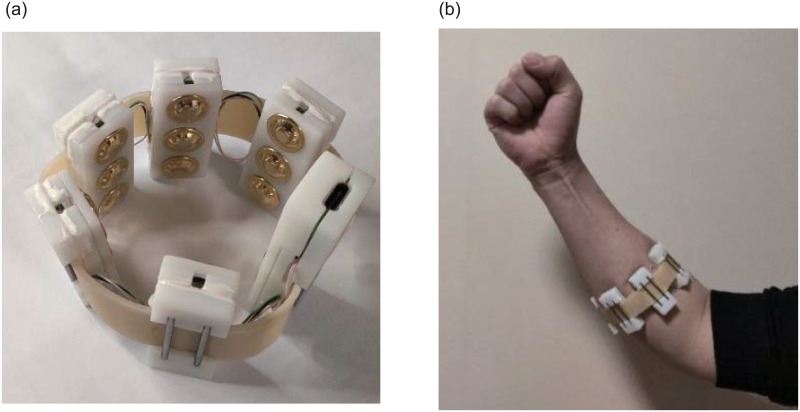
sEMG armband and its usage diagram.

**Fig 3:**
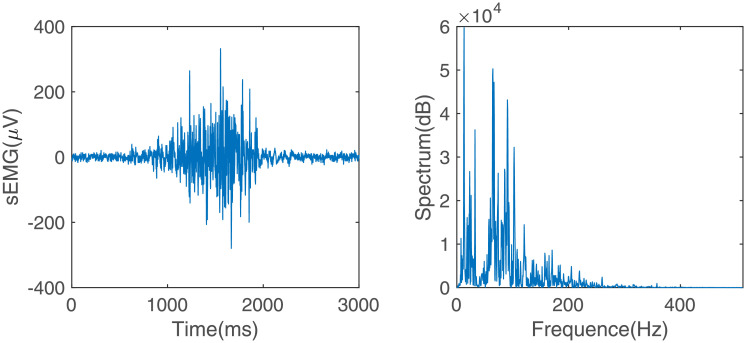
sEMG signal and its spectrum.

Fig 2 shows the armband with six sEMG sensors and its usage diagram. There are three gold-plated electrodes in each sEMG sensor. The center distance between two snap-fasteners is 2 cm. The conditioning circuit embedded in the sensor amplifies and filters the signals which are captured by the electrodes. The pass-band and voltage gain of the conditioning circuit are 10-500 Hz and 1,000, respectively. Fig 3 shows an sEMG signal captured by the sEMG sensor and the spectrum.

#### Grasp force sensor

**Fig 4:**
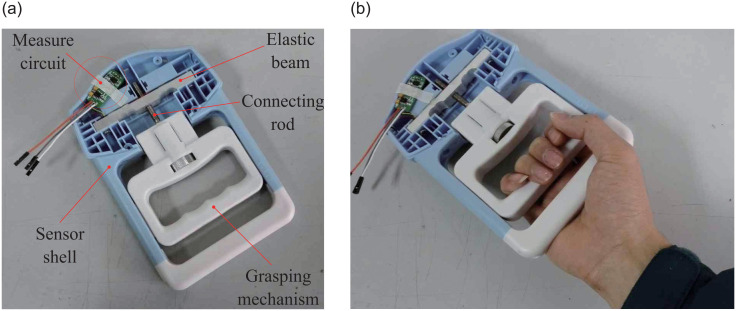
Grasp force sensor and usage diagram. (a) Grasp force sensor; (b) Usage diagram of the grasp force sensor.

Grasp force sensor and its usage diagram are shown in Fig 4. There are an elastic beam, a handle, a measurement circuit, and etc. in the grasp force sensor. Grasp force applied to the grasping mechanism will be transferred to the elastic beam through a connecting rod, so that the grasp force can be measured by attaching strain gauges to the elastic beam [24]. Table 1 shows the parameters of the the grasp force sensor.

**Table 1:** Parameters of the grasp force sensor.

Parameter	Value
Measurement range	0-60 kg
Resolution	0.1 N
Nonlinear error	0.05%FS

#### Data acquisitor and computer software

The output signals of the sEMG sensor and the grasp force sensor are digitalized at a sampling rate of 1 kHz by a data collector (USB5936) with a resolution of 12 bit. Data can be exchanged between USB5936 and computer through USB interface.

Computer software based on MFC is developed in this paper. Data captured by USB5936 can be displayed and stored by the computer software. In addition, the software can output action prompts for subjects in the experiments.

### Data acquisition

**Fig 5:**
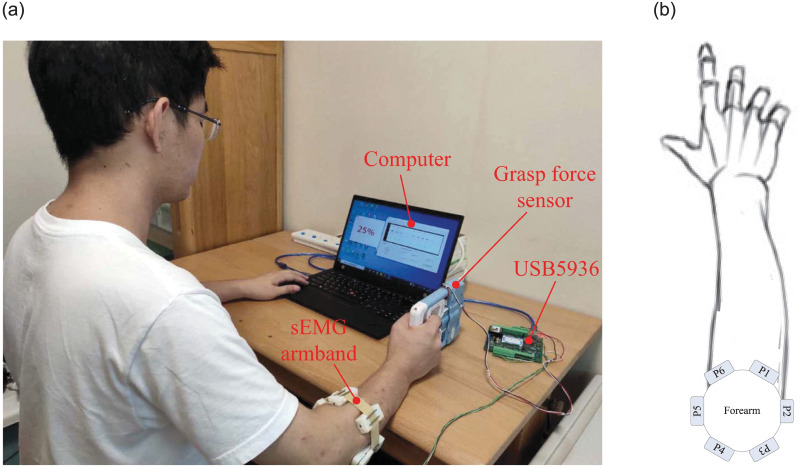
Experimental scene and distribution of measurement positions. (a) Experimental scene; (b) Measurement positions of sEMG signals.

**Fig 6:**
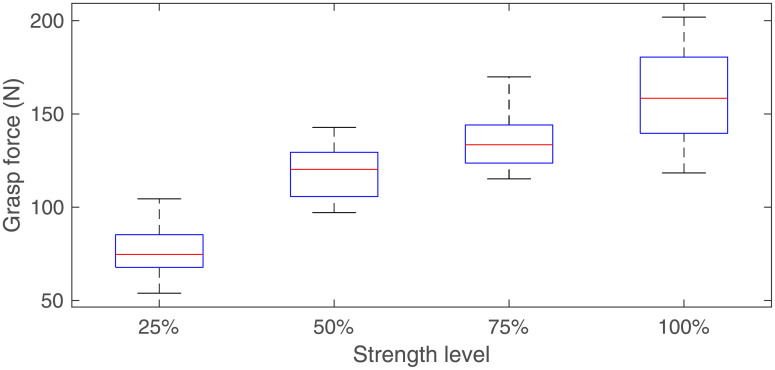
Distribution of the maximum grasp force for each action at each strength level for a subject.

**Fig 7:**
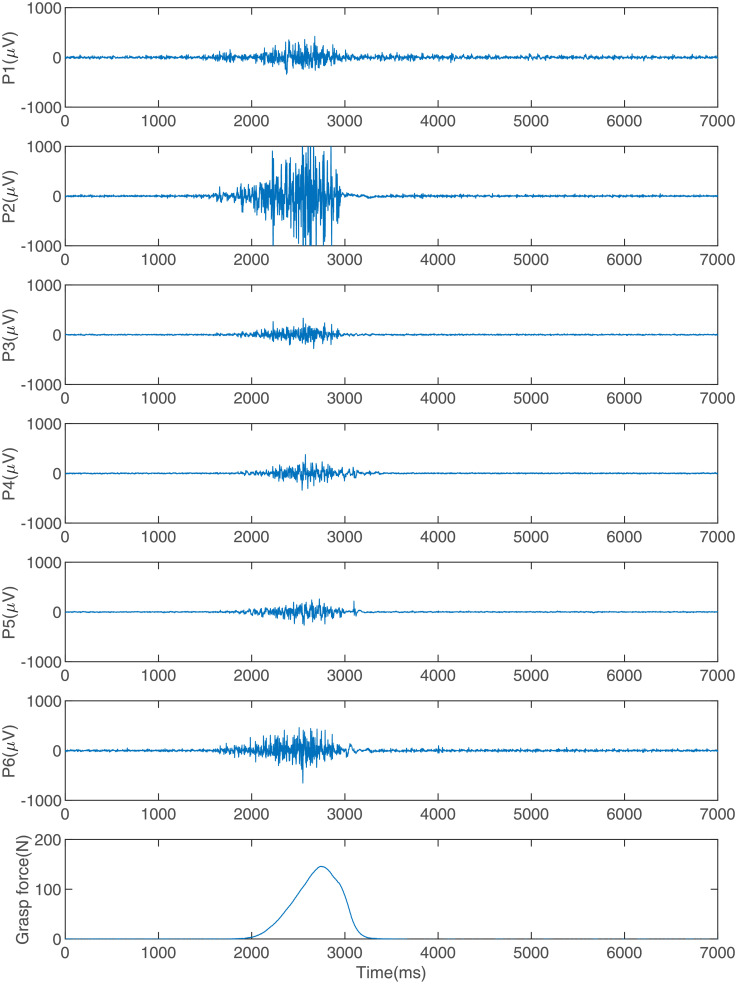
Six channels of sEMG and grasp force.

Five right hand-dominant healthy volunteers participated in the experiment were recruited from graduate students in the College of Automation, Nanjing University of Aeronautics and Astronautics. Of these, there are 4 males and 1 female (Aged from 21 to 28, mean 24.2, SD 2.77). Fig 5(a) shows the experiment scene. Subjects sat in front of a computer with their right elbow flexed at approximately 135° and with sEMG armbands on their right forearms and grasp force sensor in their right hands. Their right arms rested on the table. The paste diagram of sEMG sensors and the sEMG measuring positions are shown in Fig 5(b) and Table 2, respectively. Subjects will have 10 minutes to familiarize themselves with the experiment before experiments. In the experiments, they applied force to the grasp force sensor 60 times with roughly four different strength levels (25%, 50%, 75% and 100%) according to the prompts of the computer software. Prompt of each strength level appears 15 times. They were asked to apply force to the grasp force sensor for about one second at each action. During this procedure, sEMG and grasp force were recorded synchronously. When a subject completed a grasp action and completely relaxed his/her arm, he/she could tap the computer’s keyboard with his/her left hand finger, in which case the computer would randomly output a prompt for the next action. In total, we recorded data of 300 actions. The statistical results show that the maximum grasp forces of the five subjects are 254.9 N, 201.9 N, 291.7 N, 312.7 N, and 203.1 N, respectively. The distribution of the maximum grasp force for each action at each strength level for a subject is shown in Fig 6. Fig 7 shows six channels of sEMG and grasp force when a subject does an action.

**Table 2:** sEMG signal measurement positions.

Label	Measurement Position
P1	Extensor carpi ulnaris
P2	Flexor carpi ulnaris
P3	Flexor digitorum superficialis muscle
P4	Flexor carpi radialis
P5	Brachioradialis
P6	Extensor digitorum

The experiment mentioned above is approved by the Academic and Ethics Committee of Nanjing Tongren Hospital. All participants provided written informed consent before the start of the experiments.

**Fig 8:**
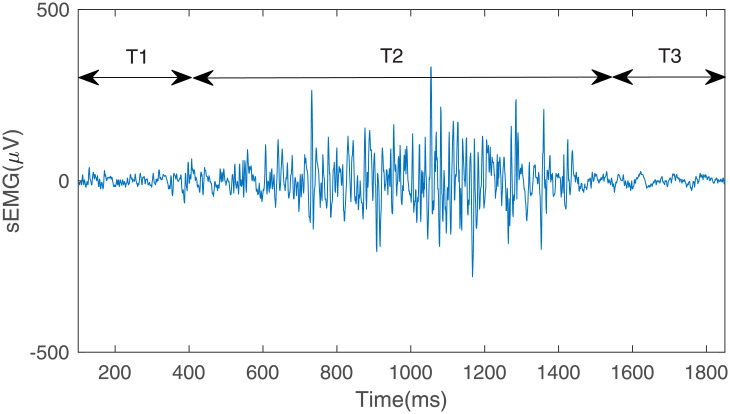
The segmented sEMG signal.

For the inconsistency of each action and its relaxation time, before feature extraction, the signals are segmented to remove the redundant data in the relaxation phase. The principle of processing is that the length of relaxation phases before and after action is one fourth of the length of the action. Fig 8 shows the segmented sEMG signal of P3 shown in Fig 7. In the figure, T1 = T3 = (1/4)T2.

### Feature extraction

In this paper, four kinds of sEMG features in time domain are extracted by using the 200 ms window with the 50% overlap.

#### Variance of sEMG (VAR)

VAR represents the power density of the sEMG signal. Its definition is as follows.

$$\begin{array}{c} VAR_{i}=\frac{1}{N}\sum_{j=i-N+1}^{i}x_{j}^{2}, \end{array}$$ (1)

where *x*_*i*_ and *x*_*i*−*n*_ are the current data and the previous *n*^*th*^ data of the sEMG signal, respectively. *N* is the length of the sliding window.

#### Zero crossing (ZC)

ZC offers frequency-domain information about sEMG signals. Its definition is as follows.

$$\begin{array}{c} ZC_{i}=\sum_{j=i-N+1}^{i}sgn\left(x_{j}x_{j-1}\right), \end{array}$$ (2)

where,$sgn\left(x\right)=\{\begin{array}{c} 1,\;\;x<0\\ 0,\;\;otherwise \end{array}$. To reduce voltage fluctuation caused by noises, restriction, |*x*_*j*_ − *x*_*j*−1_|>*threshold*, is imposed. Based on the threshold selection principle described in [25] and the sEMG sensors’ noise, the threshold is selected as 30 uV.

#### Integrated sEMG (IEMG)

IEMG depicts the power information of the sEMG. Its definition is as follows.

$$\begin{array}{c} IEMG_{i}=\sum_{j=i-N+1}^{i}\left|x_{j}\right|, \end{array}$$ (3)

#### Willison amplitude (WAMP)

WAMP reflects the level of muscle contraction. Its definition is as follows.

$$\begin{array}{c} WAMP_{i}=\sum_{j=i-N+1}^{i}f\left(x_{j}-x_{j-1}\right), \end{array}$$ (4)

where,$f\left(x\right)=\{\begin{array}{c} 1,\;\;|x|>threshold\\ 0,\;\;otherwise \end{array}$. In this paper, the threshold is selected as 150 uV according to the sEMG sensors’ gain and the presentation in [26].

In this paper, six channels of sEMG are captured and four features extracted from each channel of sEMG. We can get 63 different position sets shown in S1 Appendix by combining the six measurement positions. Similarly, combining the four sEMG features yields 15 different FSs shown in S2 Appendix. Under the condition that the same features are extracted for each channel of sEMG in the position set, we can acquire 945(15 multiplied by 63) different data sets available neural network training. Feeding these data sets to the neural networks, we can get the 945 different estimation results.

### Grasp force estimation

**Fig 9:**
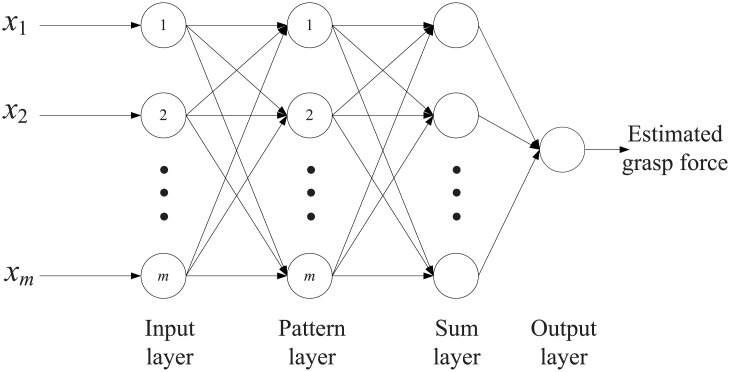
The structure of GRNN.

The purpose of force estimation is to obtain the reproducible relationship between sEMG features and the grasp force. In this paper, GRNN is introduced to estimate the grasp force. Four fifths of actions of all five subjects are randomly selected and all feature data in the selected actions are used for model training, while the remaining one fifth is used for model testing. The experiment is repeated ten times for each data set. In this way, we obtain 9,450 estimation results. Model training and testing are conducted in MATLAB. Fig 9 shows the structure of GRNN. In this paper, the input dimension of the input layer is determined by the dimension of the data sets obtained above.

### Performance evaluation

Normalized root mean square (NRMS), normalized mean of absolute error (NMAE), and correlation coefficient (CC) are introduced to evaluate the performance of grasp force estimation.

$$\begin{array}{c} NRMS=\frac{\sqrt{\frac{\sum_{i=1}^{N}{\left(\tilde{f_{i}}-f_{i}\right)}^{2}}{N-1}}}{f_{max}-f_{min}} \end{array}$$ (5)

$$\begin{array}{c} NMAE=\frac{\sum_{i=1}^{N}\left|\tilde{f_{i}}-f_{i}\right|}{N(f_{max}-f_{min})} \end{array}$$ (6)

$$\begin{array}{c} CC=\frac{cov(\tilde{f_{x}},f_{x})}{\tilde{\sigma_{f_{x}}}\sigma_{f_{x}}} \end{array}$$ (7)

where $\tilde{f_{i}}$ and *f*_*i*_ are the estimated force and the actual grasp force, respectively; *N* is the length of the data; *f*_*max*_ and *f*_*min*_ are the maximum and minium values of subjects’ grasp force captured in experiment, respectively.

A greater NMAE means poorer estimated result. A greater NRMS means a larger fluctuation in the estimated result. In addition, a greater CC means that the estimated result is similar to a larger extent to the force measured by the force sensor.

### Optimization strategy

ANOVA is employed to analyze the grasp force estimation results obtained from the above-mentioned 945 different data sets. In this paper, feature and measurement position are selected as the factors influencing estimation accuracy. Each FS and MPS represent a level for the factors of feature and measurement position, respectively. That is to say, there are 15 and 63 levels for the factors of feature and measurement position, respectively. The p-value of ANOVA results reveals the significance of the effects of different levels of factors on grasp force estimation results. Here, the significance level of p-value is set at 0.05. A p-value < 0.05 indicates that different levels of factors have a significant effect on the results. When significance is detected, Tukey HSD testing is conducted. As a result, levels of factors will be grouped into several subsets, while levels with similar effects on estimation accuracy will be grouped into one subset. We consider that in the subset with the highest estimation accuracy, the level which contains the least number of measurement positions /features is the optimal level. If there several levels meet the above requirements, the level with the highest estimation accuracy is the optimal one. We conducted ANOVA and Tukey HSD testing in IBM SPSS Statistics 19.

## Results

### Grasp force estimation based on different FSs and MPSs

**Fig 10:**
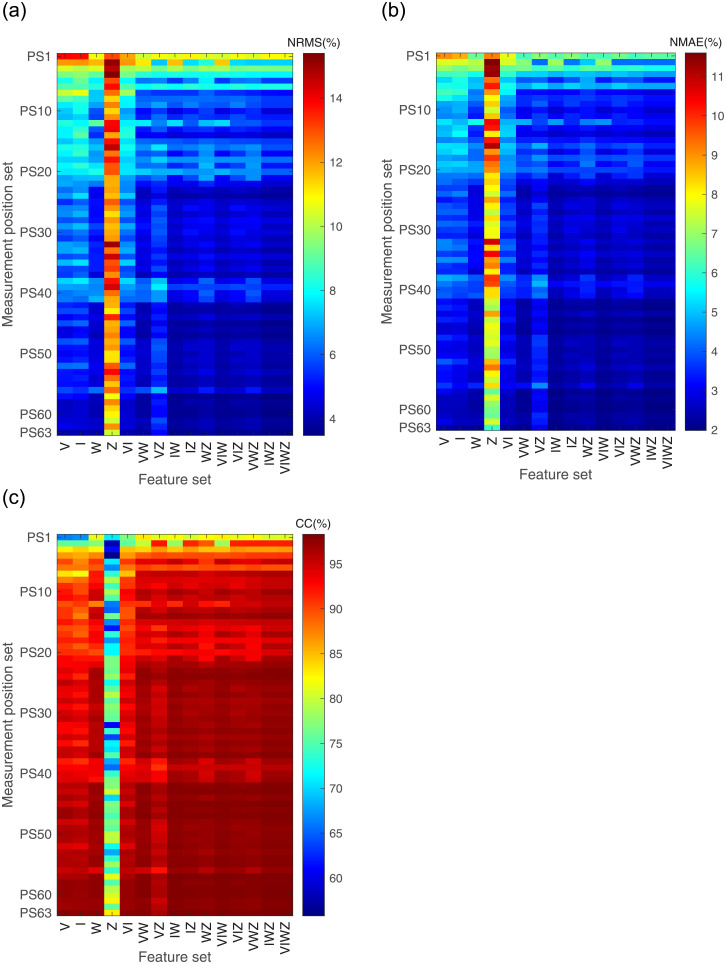
Grasp force estimation results when different FSs and MPSs are used. (a) The NRMS of the estimation error; (b) The NMAE of the estimation error; (c) The CC between actual grasp force and estimated grasp force.

Fig 10 shows the estimated results of the grasp force when different FSs and different MPSs are used. The results are significantly different. In the same FS, the estimated results based on different MPSs are different. Similarly, the estimated results based on different FSs are different in the same MPS. In addition, the results of two-way ANOVA indicate that the interaction of FS and MPS also has a significant effect on estimation results.

On the whole, with the increase of sEMG feature number or the increase of measurement position, estimation accuracy will increase as well. In particular, in cases where one sEMG feature is used for grasp force estimation, the results based on Z are significantly worse than the others. Where two sEMG features are used, the results based on VI and VZ are worse than the others.

**Fig 11:**
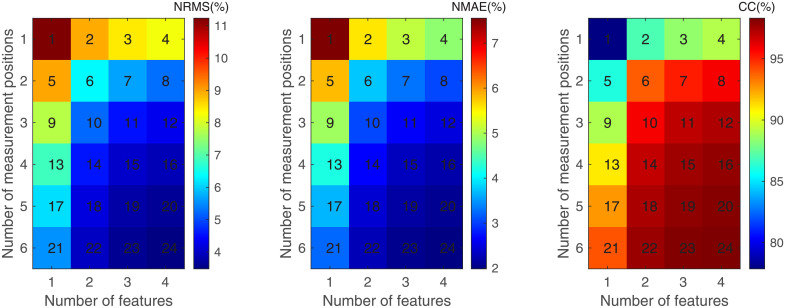
Results estimated when different numbers of measurement positions and features are employed. (a) The NRMS of the estimation error; (b) The NMAE of the estimation error; (c) The CC between estimated force and actual force.

Average estimated results are shown in Fig 11 when different numbers of measurement positions and features are employed. In the case of the same number of measurement positions, the estimation accuracy is improved with the increase of sEMG feature number. Similarly, in the case of the same number of sEMG feature number, the estimation accuracy is improved with the increase of measurement positions.

As mentioned above, the input dimension of GRNN is determined by the number of features and measurement positions. That is, the input dimension of GRNN equals to the number of features multiplied by the number of measurement positions. For different FSs and MPSs, the input dimension may be the same, such as regions 4, 6 and 13 in Fig 10. Regions 4, 6, 13 are the result of one feature and four measurement positions, two features and two measurement positions, four features and one measurement position, respectively. Although the input dimension of these three regions is 4, the numbers of features and measurement positions are different, and the results of these regions are different in all evaluation indexes. This phenomenon also occurs in input dimensions of 2, 3, 6, 8, and 12.

**Fig 12:**
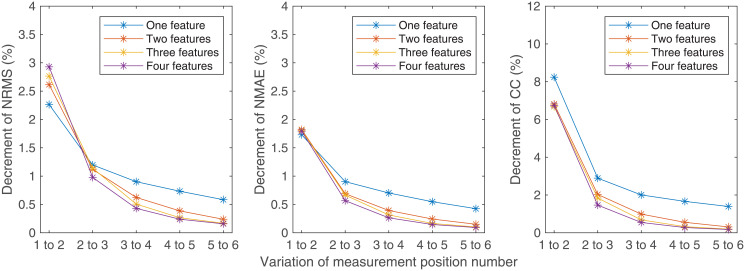
Variations of the estimated results with the variation of measurement position number. (a) The variations of NRMS; (b) The variations of NMAE; (c) The variations of CC.

**Fig 13:**
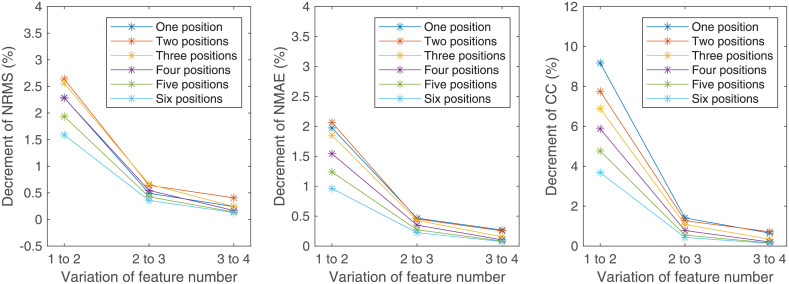
Variations of the estimated results with the variation of feature number. (a) The variations of NRMS; (b) The variations of NMAE; (c) The variations of CC.

The variations of estimated results with the variation of feature number and the variation of measurement position number are shown in Figs 12 and 13, respectively. With the increase of feature number or measurement position number, the decrement of NRMS and NMAE and the increment of CC reduce asymptotically.

**Fig 14:**
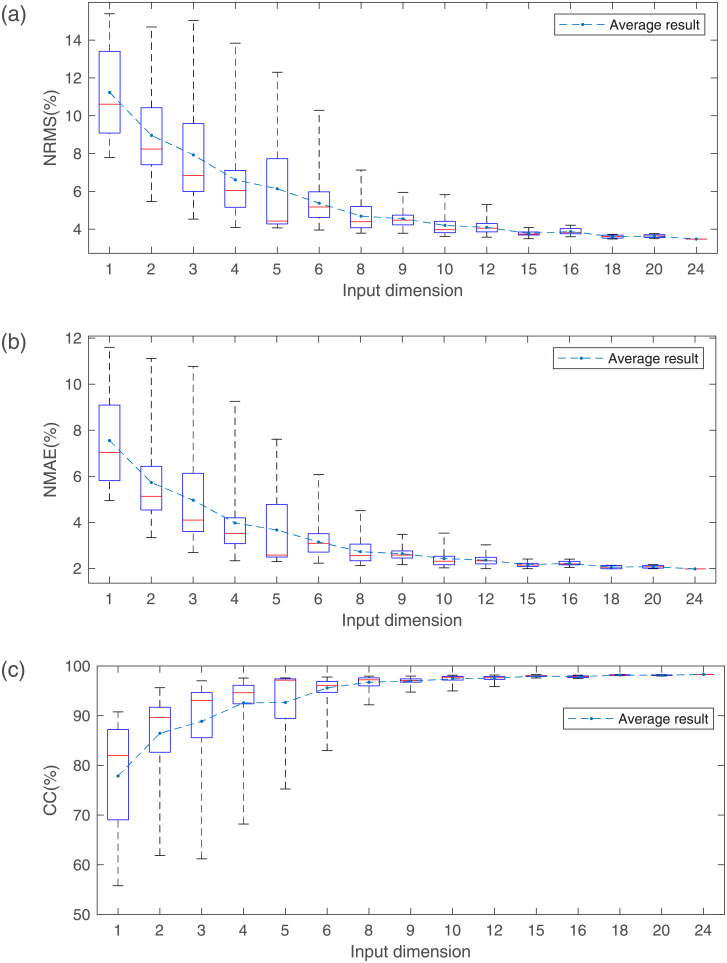
Estimated results of the grasp force when GRNNs with different input dimensions are used. (a) The NRMS of the estimation error; (b) The NMAE of the estimation error; (c) The CC between actual grasp force and estimated grasp force.

The input dimension of the network determines the computational burden directly; therefore, in this paper, the input dimension depends on the number of measurement positions and the number of features. When MPS is selected, the input dimension depends on the number of features. Similarly, when FS is selected, the input dimension depends on the number of measurement positions. We obtain the statistical estimated results when GRNNs with different input dimensions are used, as shown in Fig 14. There are significant differences in the results obtained from GRNNs with the same input dimension. The estimated results fluctuate greatly when the input dimension is small, especially less than 10. Although the average estimation accuracy shows an increasing trend with the increase in input dimension, neural networks with a small input dimension may produce a better result.

### Feature optimization for different MPSs

**Fig 15:**
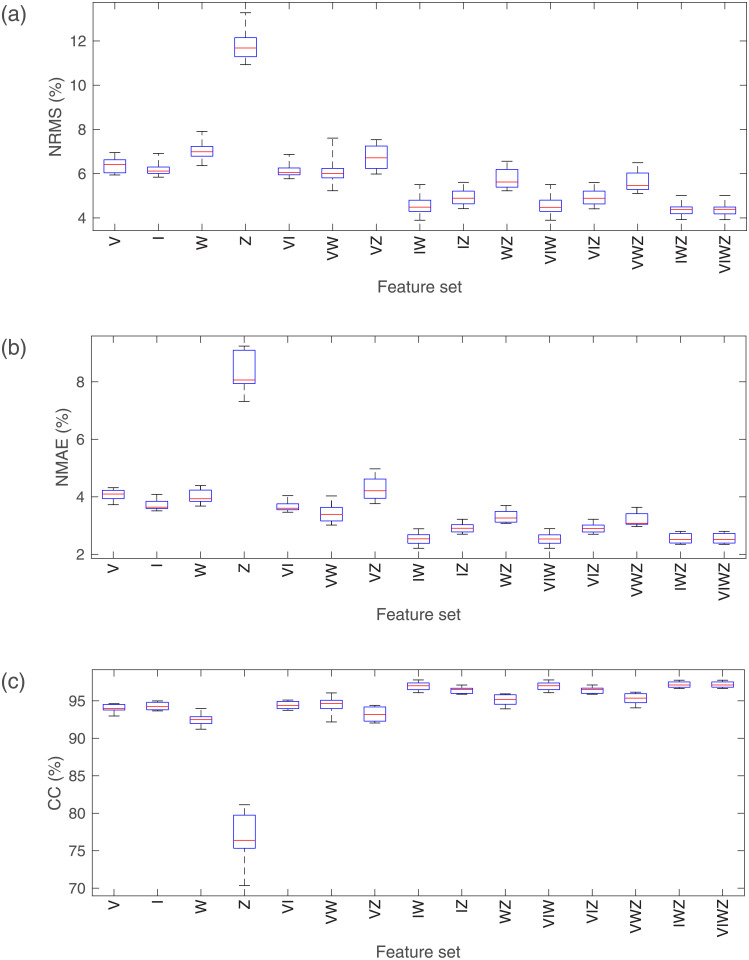
Estimated results of PS41 when different FSs are used. (a) The NRMS of the estimation error; (b) The NMAE of the estimation error; (c) The CC between actual grasp force and estimated grasp force.

Take the MPS of PS41 (the combination of P4, P5 and P6) as an example. Fig 15 shows the estimated results when different features are used. For three performance evaluation indexes, the p-values of the ANOVA are all far less than 0.05. These indicate that the effect of different FSs on the estimation results is significantly different.

Table 3 shows the Tukey HSD testing results for NRMS. Fifteen different FSs are grouped into seven subsets. There are six FSs (VIWZ, IWZ, VIW, IW, VIZ and IZ) in the subset with the highest estimation accuracy. IW is the optimal FS for the MPS of PS41 which exhibits an estimation error NRMS of 4.56% based on the optimization principle described in section 2.6. Similarly, when the focus is on the other two evaluation indexes, NMAE and CC, IW is also the optimal FS.

**Table 3:** Tukey HSD testing results for NRMS (%) of the MPS of PS41.

FS	Subset						
	1	2	3	4	5	6	7
VIWZ	4.4103						
IWZ	4.4113						
VIW	4.5638						
IW	4.5651						
VIZ	4.9456	4.9456					
IZ	4.9508	4.9508					
VWZ		5.6383	5.6383				
WZ			5.7448	5.7448			
VW			6.1065	6.1065	6.1065		
VI			6.1369	6.1369	6.1369		
I			6.2024	6.2024	6.2024		
V				6.3764	6.3764	6.3764	
VZ					6.7250	6.7250	
W						7.0598	
Z							11.8673
Sig.	0.3867	0.0739	0.3150	0.1574	0.1823	0.0835	1.0000

Table 4 shows the optimal FSs for different MPSs in different evaluation indexes. In most cases, W or IW are the optimal FS. For all three evaluation indexes, when the number of measurement positions is greater than or equal to four, W is the optimal feature in most case. Though, Z is the worst feature when one feature is used, when combined with other features, it may become the optimal FS for some MPSs.

**Table 4:** Optimal FSs for different MPSs.

MPS	Evaluation index			MPS	Evaluation index		
	NRMS	NMAE	CC		NRMS	NMAE	CC
P1	W	W	W	P2+P3+P4	IW	WZ	W
P2	VZ	VZ	VZ	P2+P3+P5	IW	IW	W
P3	VZ	IZ	VZ	P2+P3+P6	W	IW	W
P4	WZ	IW	WZ	P2+P4+P5	IW	IW	W
P5	IW	IW	IW	P2+P4+P6	IW	IW	W
P6	W	W	W	P2+P5+P6	W	IW	W
P1+P2	IZ	IW	IZ	P3+P4+P5	IW	IW	IW
P1+P3	VW	IW	VW	P3+P4+P6	IW	IW	W
P1+P4	IW	IW	IW	P3+P5+P6	IW	IW	IW
P1+P5	IW	IW	IW	P4+P5+P6	IW	IW	IW
P1+P6	W	W	W	P1+P2+P3+P4	W	W	W
P2+P3	IZ	IZ	IZ	P1+P2+P3+P5	IZ	IW	W
P2+P4	IW	IW	IW	P1+P2+P3+P6	W	W	W
P2+P5	IW	IW	IW	P1+P2+P4+P5	W	IW	W
P2+P6	IZ	IW	W	P1+P2+P4+P6	W	W	W
P3+P4	IW	IW	W	P1+P2+P5+P6	W	W	W
P3+P5	IW	IW	IW	P1+P3+P4+P5	W	W	W
P3+P6	W	W	W	P1+P3+P4+P6	W	W	W
P4+P5	IW	IW	IW	P1+P3+P5+P6	W	W	W
P4+P6	IZ	WZ	IZ	P1+P4+P5+P6	W	W	W
P5+P6	IW	IW	IW	P2+P3+P4+P5	IW	IW	W
P1+P2+P3	IZ	IW	IZ	P2+P3+P4+P6	W	W	W
P1+P2+P4	IW	IW	W	P2+P3+P5+P6	W	IW	W
P1+P2+P5	IZ	IW	W	P2+P4+P5+P6	IW	IW	W
P1+P2+P6	IW	IW	IW	P3+P4+P5+P6	IW	IW	I
P1+P3+P4	W	W	W	P1+P2+P3+P4+P5	W	W	W
P1+P3+P5	IW	IW	W				

### Measurement position optimization for different FSs

**Fig 16:**
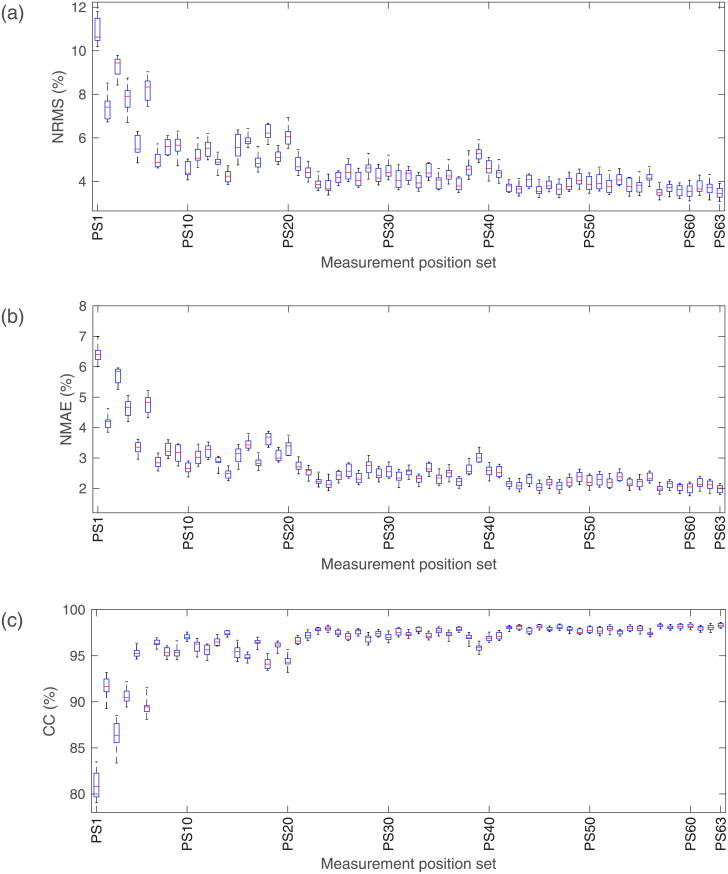
Estimated results of VIWZ when different MPSs are used. (a) The NRMS of the estimation error; (b) The NMAE of the estimation error; (c) The CC between actual grasp force and estimated grasp force.

Take the FS of VIWZ as an example. The estimated results are shown in Fig 16 when different MPSs are used. The p-values of one-way ANOVA for NRMS, NMAE and CC are all less than 0.05. That is to say, there are significant differences among the results based on 63 different MPSs in all three evaluation indexes. Similarly with the optimization of FS, Tukey HSD testing is conducted for the results of each FS, and we get the optimal MPS for different FSs as shown in Table 5.

**Table 5:** Optimal MPSs for different FSs.

FS	Evaluation index		
	NRMS	NMAE	CC
V	P1+P2+P4+P6	P1+P2+P4+P6	P1+P2+P4+P6
I	P1+P2+P3+P4+P6	P1+P2+P4+P5+P6	P1+P2+P5+P6
W	P2+P5+P6	P1+P2+P5+P6	P2+P5+P6
Z	P5+P6	P1+P5	P1+P3
VI	P1+P2+P3+P4+P6	P1+P2+P4+P5+P6	P1+P2+P4+P6
VZ	P1+P2+P5	P1+P2+P5	P1+P2+P5
IW	P2+P5	P2+P5	P1+P2
IZ	P1+P2+P5	P1+P2+P5	P2+P5
WZ	P1+P2+P5	P1+P2+P5	P1+P2+P5
VIW	P1+P2+P5	P1+P2+P5	P1+P2+P5
VIZ	P1+P2+P5	P1+P2+P5	P1+P2+P5
VWZ	P1+P2+P5	P1+P2+P5	P1+P2+P5
VWZ	P1+P2+P5	P1+P2+P5	P1+P2+P5
IWZ	P1+P2+P5	P1+P2+P5	P1+P2+P5
VIWZ	P1+P2+P5	P1+P2+P5	P1+P2+P5

For FSs with fewer features, their optimal MPS are different. With increasing number of features, the optimal MPS ultimately stabilizes at P1+P2+P5. For almost all FSs, P1 is included in their optimal MPS. For FSs containing three or four features besides WZ, their optimal MPSs are the same, i.e. P1+P2+P5, for all the three evaluation indexes.

## Conclusions

To solve the problem that increased numbers of features and measurement positions add computational burden to grasp force estimation based on sEMG and GRNN, an sEMG feature and measurement position optimization strategy is proposed following the idea of using fewer features and measurement positions to get a more desirable estimation accuracy. We conduct the experiments through capturing six channels of sEMG and extracting four time-domain features. By combining the positions and features, we obtain a series of vectors, and feed them to GRNN for grasp force estimation. Results obtained from different FSs and MPSs are analyzed by ANOVA and Tukey HSD testing. Our experimental results show that sEMG features and measurement positions significantly affect the accuracy of grasp force estimation. We obtain the optimal MPS for different FSs and the optimal FS for different MPSs. Of these, when using FS of VIWZ (contains 4 features), the optimal MPS is P1+P2+P5 (contains 3 measurement positions). When using MPS of P1+P2+P3+P4+P5+P6 (contains 6 measurement positions), the optimal FS is W (contains 1 feature). In addition, the effects of the number of measurement positions and features, the variation of the numbers of measurement positions and features, and the input dimension of GRNN on grasp force estimation results are also examined in the paper.

In the future, we will conduct further studies to investigate the feature and measurement position optimization strategy for other forms of hand force estimation.

## Supporting information

S1 Appendix sEMG signal MPSs. (PDF)

S2 Appendix sEMG FSs. (PDF)
