# Supplementary material for: Optimal strategy of sEMG feature and measurement position for grasp force estimation
Source: PLoS One. 2021 Mar 30;16(3):e0247883. doi: 10.1371/journal.pone.0247883 (PMC8009426; doi:10.1371/journal.pone.0247883)
Supplement: S1 Appendix — (PDF) [file pone.0247883.s001.pdf]

S1 Appendix. sEMG Signal MPSs.

Label of MPS	Combination of measurement positions	Label of MPS	Combination of measurement positions
PS1	P1	PS33	P2+P3+P5
PS2	P2	PS34	P2+P3+P6
PS3	P3	PS35	P2+P4+P5
PS4	P4	PS36	P2+P4+P6
PS5	P5	PS37	P2+P5+P6
PS6	P6	PS38	P3+P4+P5
PS7	P1+P2	PS39	P3+P4+P6
PS8	P1+P3	PS40	P3+P5+P6
PS9	P1+P4	PS41	P4+P5+P6
PS10	P1+P5	PS42	P1+P2+P3+P4
PS11	P1+P6	PS43	P1+P2+P3+P5
PS12	P2+P3	PS44	P1+P2+P3+P6
PS13	P2+P4	PS45	P1+P2+P4+P5
PS14	P2+P5	PS46	P1+P2+P4+P6
PS15	P2+P6	PS47	P1+P2+P5+P6
PS16	P3+P4	PS48	P1+P3+P4+P5
PS17	P3+P5	PS49	P1+P3+P4+P6
PS18	P3+P6	PS50	P1+P3+P5+P6
PS19	P4+P5	PS51	P1+P4+P5+P6
PS20	P4+P6	PS52	P2+P3+P4+P5
PS21	P5+P6	PS53	P2+P3+P4+P6
PS22	P1+P2+P3	PS54	P2+P3+P5+P6
PS23	P1+P2+P4	PS55	P2+P4+P5+P6
PS24	P1+P2+P5	PS56	P3+P4+P5+P6
PS25	P1+P2+P6	PS57	P1+P2+P3+P4+P5
PS26	P1+P3+P4	PS58	P1+P2+P3+P4+P6
PS27	P1+P3+P5	PS59	P1+P2+P3+P5+P6
PS28	P1+P3+P6	PS60	P1+P2+P4+P5+P6
PS29	P1+P4+P5	PS61	P1+P3+P4+P5+P6
PS30	P1+P4+P6	PS62	P2+P3+P4+P5+P6
PS31	P1+P5+P6	PS63	P1+P2+P3+P4+P5+P6
PS32	P2+P3+P4		
