# Supplementary material for: Optimal strategy of sEMG feature and measurement position for grasp force estimation
Source: PLoS One. 2021 Mar 30;16(3):e0247883. doi: 10.1371/journal.pone.0247883 (PMC8009426; doi:10.1371/journal.pone.0247883)
Supplement: S2 Appendix — (PDF) [file pone.0247883.s002.pdf]

S2 Appendix. sEMG FSs.

Label of FS	Combination of sEMG features	Label of FS	Combination of sEMG features
V	VAR	IZ	IEMG+ZC
I	IEMG	WZ	WAMP+ZC
W	WAMP	VIW	VAR+IEMG+WAMP
Z	ZC	VIZ	VAR+IEMG+ZC
VI	VAR+IEMG	VWZ	VAR+WAMP+ZC
VW	VAR+WAMP	IWZ	IEMG+WAMP+ZC
VZ	VAR+ZC	VIWZ	VAR+IEMG+WAMP+ZC
IW	IEMG+WAMP		
